# Suppressed ion-scale turbulence in a hot high-β plasma

**DOI:** 10.1038/ncomms13860

**Published:** 2016-12-21

**Authors:** L. Schmitz, D. P. Fulton, E. Ruskov, C. Lau, B. H. Deng, T. Tajima, M. W. Binderbauer, I. Holod, Z. Lin, H. Gota, M. Tuszewski, S. A. Dettrick, L. C. Steinhauer

**Affiliations:** 1 Department of Physics and Astronomy, University of California Los Angeles, Los Angeles, California 90095, USA; 2 Tri Alpha Energy, Inc., P.O. Box 7010, Rancho Santa Margarita, California 92688, USA; 3 Department of Physics and Astronomy, University of California, Irvine, Irvine, California 92697, USA

## Abstract

An economic magnetic fusion reactor favours a high ratio of plasma kinetic pressure to magnetic pressure in a well-confined, hot plasma with low thermal losses across the confining magnetic field. Field-reversed configuration (FRC) plasmas are potentially attractive as a reactor concept, achieving high plasma pressure in a simple axisymmetric geometry. Here, we show that FRC plasmas have unique, beneficial microstability properties that differ from typical regimes in toroidal confinement devices. Ion-scale fluctuations are found to be absent or strongly suppressed in the plasma core, mainly due to the large FRC ion orbits, resulting in near-classical thermal ion confinement. In the surrounding boundary layer plasma, ion- and electron-scale turbulence is observed once a critical pressure gradient is exceeded. The critical gradient increases in the presence of sheared plasma flow induced via electrostatic biasing, opening the prospect of active boundary and transport control in view of reactor requirements.

## Abstract (web summary)

Magnetic fusion reactors with higher ratio of plasma kinetic pressure to magnetic pressure are economically desirable. The authors demonstrate a path to such a reactor in a field reversed configuration that can attain microstability and reduced particle and thermal fluxes by manipulating the shear flow.

Magnetic confinement fusion offers the prospect of a carbon-neutral, environmentally responsible and inexaustible energy source. The present main-line approach to magnetic fusion energy is via the tokamak concept^1^ relying on a strong toroidal (doughnut-shaped) magnetic field to confine plasma at temperatures characteristic of the interior of stars (∼100 million °K). The hydrogen isotopes deuterium (D) and tritium (T) fuse at these temperatures, releasing energy mainly in the form of neutrons. Fusion energy is converted to thermal energy in a blanket surrounding the plasma, and must be recovered via a thermal cycle. The most advanced tokamak to date, the International Thermonuclear Experimental Reactor, is presently under construction, to test and demonstrate the sustained production of fusion energy for the first time.

The field-reversed configuration^2^,^3^ (FRC) is characterized by a much higher ratio *β* of the plasma kinetic pressure to the external magnetic field energy density, with volume-averaged *β*≤0.9 and peak *β*_max_=1. This magnetic configuration is of great interest as a fusion reactor concept due to its compact, axisymmetric geometry and the potential for aneutronic fusion based on advanced fuels, such as the proton-boron fusion reaction^4^ (p-B^11^). Other important advantages of the FRC concept include the presence of a natural divertor which provides for efficient exhaust of fusion products, and also allows for a large radial expansion of the magnetic flux. In turn the particle and heat outflux from the FRC core plasma is distributed over a larger surface area, and consequently the heat load to plasma-facing components is substantially reduced.

FRCs are also a powerful tool for fundamental plasma research, and allow investigating the properties and stability of high *β*, high temperature collisionless plasmas in a laboratory environment. Such conditions are ubiquitous in astrophysical plasmas, for example in accretion discs^5^,^6^, in the outer solar corona^6^, in solar mass ejections^7^, and in stellar superflares^7^. In particular, the physics of magnetic reconnection, and the associated transfer of magnetic energy to kinetic (particle) energy is actively researched using FRC plasmas.

In virtually all magnetic confinement devices including FRCs^2^,^3^ as well as in astrophysical plasmas, anomalously large plasma resistivity, and plasma energy and particle transport in excess of classical collisional transport perpendicular to the magnetic field have been observed. Long wavelength turbulence, with wavelengths larger than the ion gyration (Larmor) radius *ρ*_i_, is typically most detrimental to plasma confinement. Due to the high ion energy and the comparatively low magnetic field, FRC plasmas are characterized by large ion Larmor radii *ρ*_i_ of the thermal ion population, and correspondingly high ρ*=*ρ*_i_/*R*∼0.1, where *R* is the characteristic magnetic field reversal (null field) radius. Hence FRC turbulence properties can be substantially different^8^,^9^,^10^,^11^,^12^ from tokamak plasmas, where ρ*∼10^−3^−10^−2^, and where ion-temperature-gradient-driven instabilities are often dominant, with perpendicular wavelengths substantially larger than the ion Larmor radius (*k*_⊥_*ρ*_i_<1, where *k*_⊥_is the turbulence wavenumber perpendicular to the magnetic field).

Direct observation or systematic identification of the turbulent processes that influence or limit confinement has been lacking so far in FRC plasmas. Earlier work has focused on anomalous resistivity and transport due to the Lower Hybrid Drift Instability^13^ but an experimental investigation of short wavelength turbulence via microwave forward scattering did not show sufficiently large fluctuation levels^14^. Short wavelength turbulence driven by the radial electron temperature gradient^15^ (ETG modes) as well as other driftwave instabilities^16^ have also been postulated theoretically for specific ratios of density and electron/ion temperature gradient scale lengths, but not confirmed experimentally to date in FRC plasmas.

In this paper we show that, in contrast to tokamaks, long wavelength, ion-scale modes (*k*_⊥_*ρ*_i_<1) are stable or substantially reduced in the FRC core of the C-2 device^17^,^18^ (Fig. 1), resulting in an inverted toroidal wavenumber spectrum. This observation is consistent with essentially classical ion thermal confinement, with the radial ion thermal diffusivity 
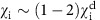
 evaluated from 1-D and 2-D transport analysis^19^, where 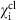 is the classical collisional ion thermal diffusivity. Finite ion Larmor radius (FLR) effects^9^,^10^,^11^,^12^ are found to contribute crucially to the observed stability of long wavelength modes, as confirmed by gyrokinetic stability calculations^20^,^21^. The scrape-off layer (SOL) plasma is found to be unstable to multiscale drift-interchange modes (0.5<*k*_*θ*_*ρ*_s_≤40, where *ρ*_s_ is the ion sound Larmor radius 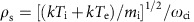, and *ω*_ci_ is the ion cyclotron frequency, due to large radial density/temperature gradients in conjunction with the (moderate) field line curvature^22^. A critical density/electron pressure gradient has to be exceeded for instability near the separatrix and in the SOL. Finally, evidence for a radial transport barrier due to **E** × **B** flow shear in the SOL is presented. This observation is very significant in that it affects the particle and thermal fluxes across the FRC separatrix, and hence plays a decisive role in overall FRC particle and thermal confinement.

## Results

### Experimental set-up and diagnostics

**Figure 1:**
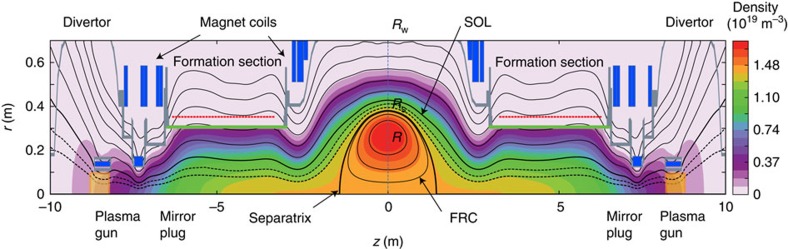
Schematic cross-section of the C-2 field-reversed configuration. Axial cross-section of the Tri Alpha Energy (TAE) field-reversed configuration (FRC) device C-2, showing the magnetic field configuration (indicated by magnetic field lines in black), and plasma density (overlayed colour contours; the colour scale bar indicates the plasma density in units of 10^19^ m^−3^) from a two-dimensional magneto-hydrodynamic (MHD) equilibrium calculation using the LamyRidge code^24^. *r* and *z* are the radial and axial coordinates. The null field radius (*R*), separatrix radius (*R*_s_), and wall radius *R*_W_=0.7 m of the device are indicated. The separatrix location is indicated by a solid black line. The width of a flux tube at the thermal ion gyroradius in the scrape-off layer (SOL) is indicated by two dashed field lines. The mirror and mirror plug magnetic field coils are shown in blue; the solenoidal field coils in the confinement and formation sections, located at larger radii, are not shown here. C-2 has two formation sections and two divertors as indicated on opposite sides of the central confinement vessel. The locations of the plasma guns and mirror plug coils are also indicated.

The C-2 FRC plasma is created via injecting and merging of two preformed, compact high-*β* plasmoids into a central confinement chamber with wall radius *R*_W_=0.7 m and length *L*=4.5 m, with an external solenoidal magnetic field *B*_e_=0.05–0.14 T. The magnetic configuration is shown in Fig. 1 and described in detail in the Methods section below and elsewhere^18^,^23^,^24^. Typical line-averaged plasma densities in the experiments described here are 〈*n*_e_〉=2–4 × 10^19^ m^−3^, and the ion and electron temperatures in the FRC core are *T*_i_∼400–600 eV and *T*_e_∼80–130 eV. The FRC null field radius is *R*∼0.25–0.35 m in the first shot phase (*t*≤2 ms). The FRC separatrix radius is approximated via the measured excluded flux radius *R*_s_ (*R*_s_∼0.35–0.45 m initially). In the C-2 FRC, neutral beam injection (NBI, beam voltage and power *V*_B_=20 kV, *P*_B_∼4 MW) is used to extend the FRC pulse length to∼5 ms. The (closed flux surface) FRC plasma in C-2 is surrounded by an (open fieldline) mirror-confined SOL plasma. Annular washer plasma guns^25^ with inner diameter 0.11 m and outer diameter 0.13 m, located in the divertor chambers, are used for active plasma boundary control. The plasma guns produce an electrically biased plasma on open field lines in the divertor, resulting in a negative (inwards pointing) radial electric field mapping to the SOL region near the machine midplane and just outside the FRC separatrix^26^,^27^ (at radius *r*≥*R*_s_) as discussed in more detail below in the Methods section.

**Figure 2:**
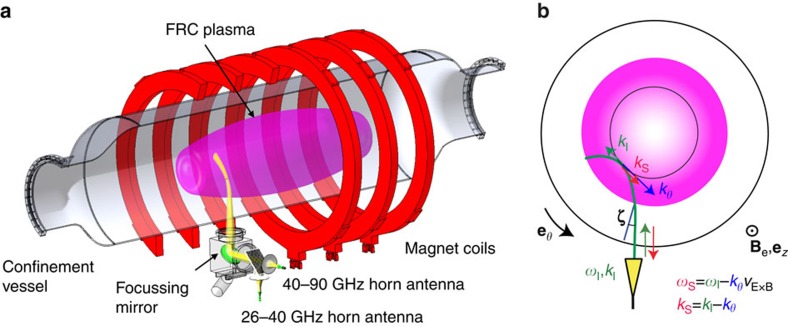
Schematic of the Doppler backscattering diagnostic. (**a**) A view of the Doppler backscattering^28^,^29^,^30^ (DBS) microwave diagnostic beam optics components and beam path. Monostatic beam optics (collocated launch and receive beam optics) is used to launch six separate microwave frequencies via a beam combiner (polarizer) and an adjustable parabolic focusing mirror. The resulting common beam trajectory is indicated by yellow shading. Frequencies in the range 26–40 GHz are launched in X-mode polarization, while frequencies above 40 GHz are launched in O-mode polarization. Gaussian beams are launched into the plasma at an oblique angle *ζ* in the toroidal plane. (**b**) toroidal plane cross-section illustrating DBS launched/backscattered beam trajectory and the probed toroidal wavenumber (for one frequency channel); the relations between the launched and backscattered frequency and wavenumber, and the probed density fluctuation wavenumber are shown. The toroidal direction and the direction of the external magnetic field **B**_e_ are also indicated.

The fluctuation data reported here is acquired via a multichannel microwave scattering diagnostic described in more detail below. Doppler Backscattering^28^,^29^,^30^ (DBS) measures the density fluctuation level at different plasma radii in the FRC core and in the SOL. Microwave radiation at six separate tunable frequencies 26 GHz<*f*<65 GHz is launched via two lensed horn antennas and quasi-optically transmitted to the plasma (Fig. 2). Backscattering by plasma density fluctuations occurs preferentially near the plasma cutoff layer for each launched frequency^29^ according to the selection rules *k*_S_=−*k*_I_=*k*_*θ*_/2 and *ω*_S_=*ω*_I_−*ν*_t_*k*_*θ*_ where the indices I and S denote the incident and backscattered wave (Fig. 2b). *ν*_t_=*ν*_E × B_+*ν*_ph_ is the toroidal fluctuation advection velocity (*v*_ph_ is the fluctuation phase velocity in the plasma frame and can be neglected here). *k*_*θ*_ is the probed resonant density fluctuation toroidal wavenumber. Variation of the toroidal launch angle allows acquiring the density fluctuation level versus toroidal wavenumber.

### Fluctuation properties and wavenumber spectra

**Figure 3:**
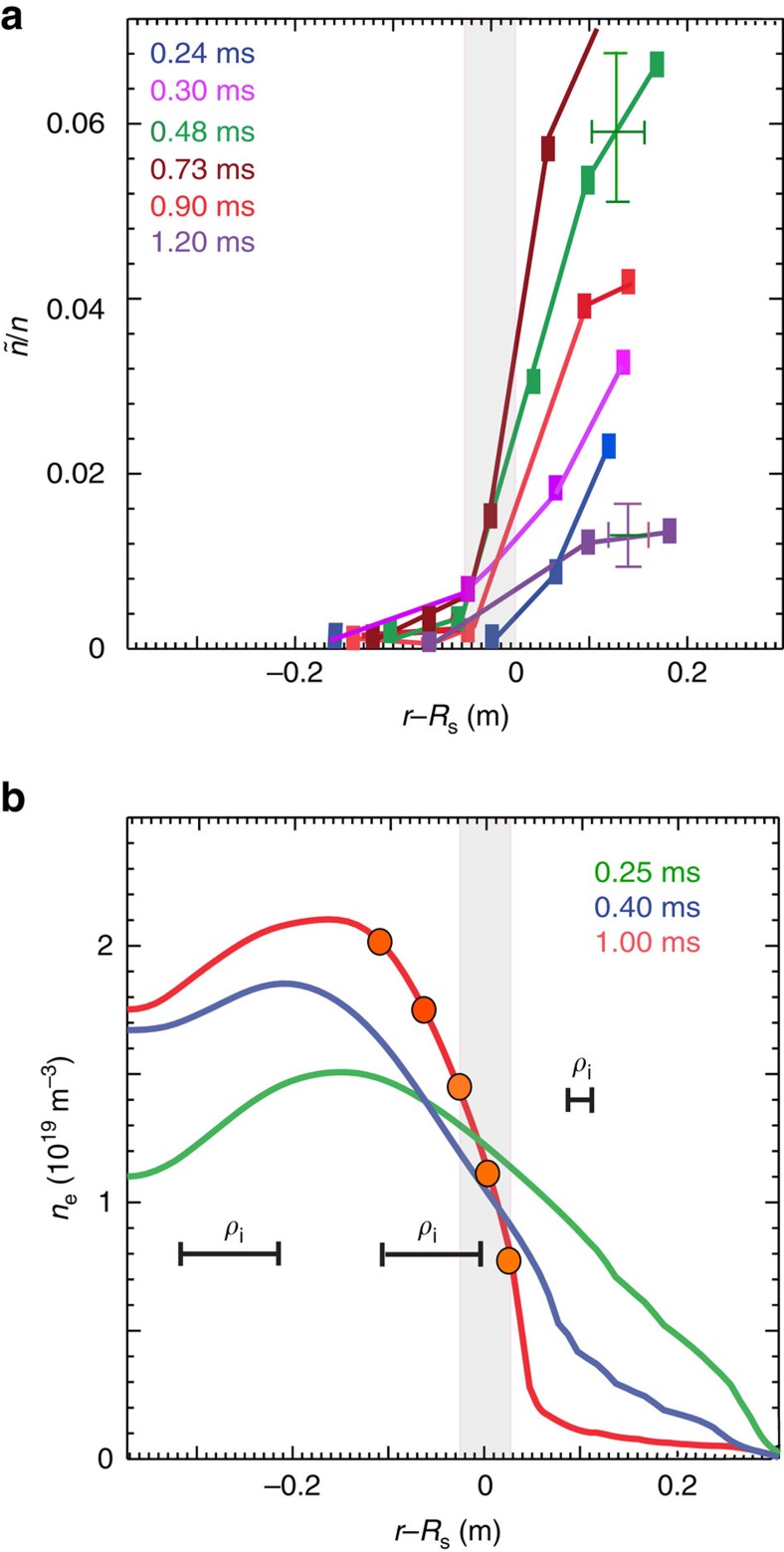
Radial profiles of the normalized density fluctuation level and plasma density. (**a**) Radial profile of the normalized density fluctuation level *ñ*/*n* at different times after compact toroid (CT) merging (*t*=0). The fluctuation levels measured via Doppler Backscattering^28^,^29^,^30^ have been calibrated via comparison to Far Infrared Scattering (FIR) data^54^. The error bars represent the standard variation (s.d.) of the measurements. (**b**) Plasma density profile measured via carbon dioxide (CO_2_) laser interferometry^53^ at three different times *t*. Typical Doppler Backscattering (DBS) probing radii (cutoff layer locations) are indicated by orange circles. The size of the thermal ion gyroradius, averaged along the field line length, is indicated at different locations in the closed fieldline region and in the scrape-off layer (SOL).

Figure 3 shows radial profiles of the normalized density fluctuation level *ñ*/*n* measured via DBS and the radial plasma density profile at different times during the discharge. Typical DBS probing positions are indicated via orange markers in Fig. 3b. The measured fluctuation toroidal wavenumber range (determined by the beam launch angle *ζ* and the plasma geometry) is centred here around *k*_*θ*_∼2–3 cm^−1^ (*k*_*θ*_*ρ*_s_∼6–9) with a spread Δ*k*_*θ*_/*k*_*θ*_∼0.9–1.2 mainly due to toroidal plasma curvature^30^,^31^. Fluctuation levels are initially low in the core and also relatively low in the SOL. SOL fluctuations increase substantially in amplitude concomitantly with the density gradient (*t*=0.4 ms), as examined in detail below. FRC core fluctuations remain low throughout the discharge.

**Figure 4:**
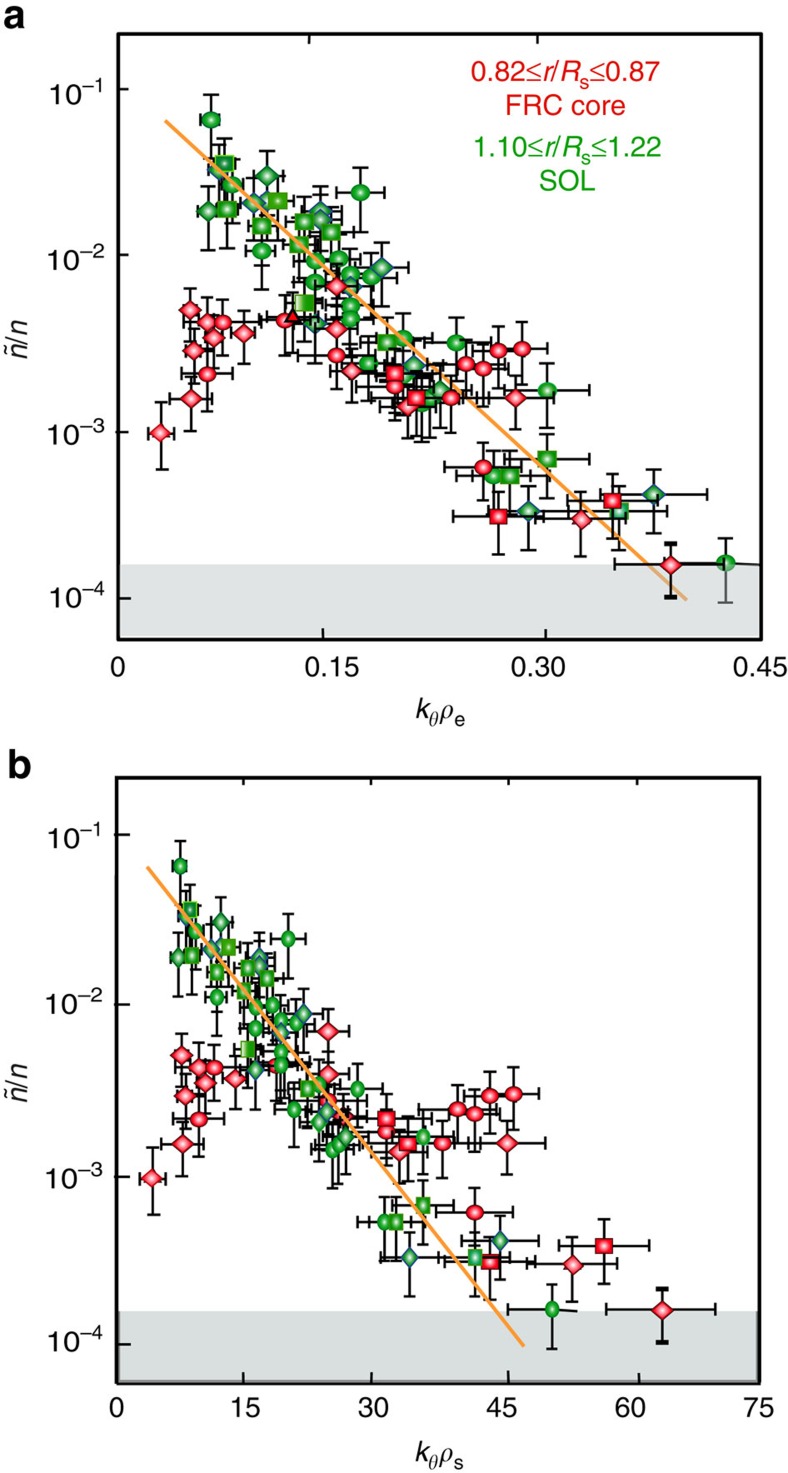
The toroidal wavenumber spectrum of density fluctuations measured via Doppler Backscattering. (**a**) Normalized density fluctuation *ñ/n* level versus toroidal wavenumber normalized to the electron gyroradius *k*_*θ*_*ρ*_e_ in the field reversed configuration (FRC) core inside the separatrix and in the scrape-off layer (SOL) (shots #29587–29610; #29750–29802). *ρ*_e_ is the electron Larmor radius. The Doppler Backscattering^28^,^29^,^30^ sensitivity limit is indicated in the figure (grey bar). (**b**) measured toroidal wavenumber spectra in the core plasma and in the scrape-off layer, plotted versus toroidal wavenumber normalized to the ion sound gyroradius *k*_*θ*_*ρ*_s_. An inverted core wavenumber spectrum is observed for *k*_*θ*_*ρ*_e_<0.05 (*k*_*θ*_*ρ*_s_<7) indicating that long wavelength (ion) modes are not present. The SOL spectrum shows the highest fluctuation levels at low wavenumber, and the fluctuation level decays exponentially with increasing wavenumber. The error bars represent the typical standard deviation (s.d.) of the measurements. The fluctuations observed in the C-2 FRC core are qualitatively consistent with low frequency (*ω*<*ω*_ci_, where *ω*_ci_ is the ion cyclotron frequency) electron drift/interchange modes (with a toroidal wavenumber range 0.05≤*k*_*θ*_*ρ*_e_≤0.45).

We now present evidence that the nature of the saturated fluctuation spectrum is entirely different in the FRC core and SOL, and also that FRC core fluctuations are fundamentally different from tokamak core plasma turbulence. Figure 4a shows the toroidal wavenumber spectra, measured just inside the FRC separatrix via DBS, and in the SOL. Fluctuation levels are plotted here versus the toroidal wavenumber normalized by the electron gyro-radius.

An inverted toroidal wavenumber spectrum is clearly observed in the FRC core, with fluctuation levels peaking at wavenumbers in the electron mode range (0.05≤*k*_*θ*_*ρ*_e_≤0.2, where 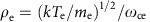 is the electron gyroradius), and *k*_*θ*_*ρ*_s_≥5. The local ion-to electron temperature ratio is *T*_i_/*T*_e_∼6.8 as determined from Doppler spectroscopy and Thomson scattering. The core spectrum extends to *k*_*θ*_*ρ*_e_≥0.4 and falls off steeply at low *k*_*θ*_*ρ*_e_. It is clear that the integrated fluctuation level is substantially lower in the core, compared to the SOL. This is a strong experimental indication that ion-scale modes are absent in the FRC core, and electron modes are dominant. The spectral peak may be shifted with respect to the linearly most unstable modes; however, the spectrum clearly indicates that longer wavelength modes are subdominant or stable. This wavenumber spectral shape profoundly affects both ion and electron thermal transport, which is also predominantly carried by low-*k* (ion-scale) fluctuations. The measured spectral shape sharply differs from wavenumber spectra measured in the core of tokamak low confinement mode (L-mode) plasmas and linear confinement devices, where the fluctuation amplitude typically decreases monotonically with increasing normalized wavenumber^32^,^33^. We can conjecture that the large ion Larmor radius in the FRC core region stabilizes ion-scale modes^9^,^10^,^11^. This conjecture will be examined further below.

The SOL spectrum, on the other hand, peaks at low *k*_*θ*_*ρ*_e_ and decays exponentially with increasing wavenumber, with a decay constant of *α*_k_∼0.16 (the corresponding exponent for the decay of the fluctuation energy density, proportional to (*ñ*/*n*)^2^, is ∼0.33). Here, the local electron- to ion temperature ratio is *T*_i_/*T*_e_∼3.7. The observed fluctuation power density at the lowest measured wavenumbers is more than two orders of magnitude higher than in the FRC core. Exponential spectra have been observed in linear and toroidal confinement devices, for example in the core of tokamak low confinement mode (L-mode) plasmas^32^,^33^. These spectra are however dominated by much lower normalized (poloidal) wavenumbers (*k*_*θ*_*ρ*_s_≤2), consistent with dominant ion-temperature-gradient and/or trapped electron mode turbulence. In contrast, the FRC SOL spectrum measured here via DBS shows substantial fluctuation levels at higher *k*_*θ*_*ρ*_s_, illustrating that fluctuations with sub-ion-Larmor-radius scales are important also outside the FRC separatrix. Notably, the core and SOL spectra roughly overlap at high *k*_*θ*_*ρ*_e_≥0.17 and both decay roughly exponentially.

Figure 4b shows the same data plotted versus the toroidal wavenumber normalized by the ion sound gyro-radius. Marked differences between the SOL and FRC core spectra are now obvious at high normalized wavenumber. This discrepancy may indicate that, as expected, electron-range modes and electron-range physics dominate at high toroidal wavenumber both in the FRC core and in the SOL.

**Figure 5:**
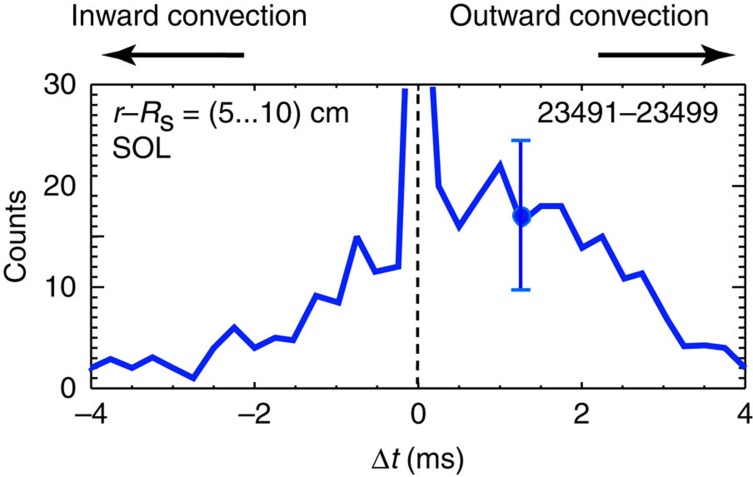
Radial propagation delay of turbulent structures in the scrape-off layer. The correlation delay of turbulent density fluctuations between two radially spaced Doppler backscattering probing locations^28^,^29^,^30^,^31^ is measured. The statistical distribution of the radial correlation delay of density fluctuations in the scape-off layer (SOL) is depicted. Positive correlation delay corresponds to a radial outward propagation of turbulent structures. The statistically averaged correlation delay Δ*t*∼1 μs indicates an average radial velocity of turbulent structures *ν*_tr_∼1−1.5* × *10^4^ m s^−1^. Shot numbers for the data are indicated. The error bar represents the typical standard deviation (s.d.) of the measurements.

We have examined the radial cross-correlation of SOL density fluctuations/turbulent structures to confirm that the observed SOL turbulence is indeed responsible for radial plasma transport. Figure 5 shows the probability distribution of the radial correlation delay, obtained from a pair of DBS channels probing two closely spaced radii (the channel spacing varies from Δ*r*∼1.5 cm early in the discharge to Δ*r*∼1 cm at later times). Data from nine FRC shots with similar parameters and similarly well-centred plasma have been averaged. Outward propagation of turbulent structures is clearly observed, with an average propagation delay of Δ*t*∼1 *μ*s, and provides strong evidence for fluctuation-driven convective radial particle/energy transport. The average radial propagation velocity of turbulent structures can be estimated as *ν*_t*r*_∼Δ*r*/Δ*t*∼1–1.5 × 10^4^ m s^−1^. The peak in the correlation delay around Δ*t*=0 is related to macroscopic plasma movement or ‘wobble' due to residual large-scale *n*=1 MHD mode activity.

### Gyrokinetic stability analysis

FRC plasmas present a considerable challenge for microstability analysis due to the large ion Larmor radii and high *β*. In particular, rigorous stability calculations for the FRC core plasma would require a fully kinetic approach, as bulk thermal ion gyroradii are comparable to the local temperature and density gradient lengths. Modelling efforts using a fully kinetic code are presently underway. As an initial step, the linear instability growth rate of modes driven by the radial density gradient and electron/ion temperature gradients have been calculated with the Gyrokinetic Toroidal Code^34^,^35^ (GTC). Originally designed to investigate and predict turbulence properties and radial thermal/particle transport fluxes in tokamaks, with predominant toroidal magnetic field, the GTC code has been adapted to the FRC geometry with purely poloidal magnetic field, using Boozer coordinates^36^ and a reformulated Poisson solver. FRC plasma equilibria produced with the LamyRidge code^24^ are first transformed from cylindrical coordinates to magnetic flux coordinates and further to Boozer coordinates. The modified GTC code has been extensively tested for convergence, as described in detail elsewhere^20^. Linear growth rate calculations based on local (flux tube) simulations (*k*_*r*_<<*k*_*θ*_) are reported here. In these calculations, modes that may exist very close to the null field radius *R* are neglected due to difficulties simulating the region of vanishing magnetic field. We note also that in the present study our local simulations separate the FRC Core and SOL plasma domains. The potential coupling between these domains will be investigated in future work.

It is important to point out that FLR effects, important due to the high bulk ion temperature, are retained in the gyrokinetic approximation via toroidal gyro-averaging. Gyro-particles sampling different locations within the gyro-ring are implemented via Bessel functions *J*_0_(*k*_*θ*_*ρ*_i_) where *ρ*_i_=*m*_*i*_*ν*_i⊥_/*eB* signifies the gyro-particle gyroradius at the instantaneous perpendicular velocity *ν*_i⊥_. For the local simulations described here, no radial gyro-averaging is performed as *k*_*r*_
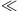
*k*_*θ*_. It is well known that the parameter ρ* (the ratio of the ion gyroradius to the characteristic radial system dimension) is important for the transport scaling obtained with nonlinear gyrokinetic simulations in toroidal devices^37^,^38^,^39^. In particular, Bohm-like transport scaling has been obtained with global simulations for small system sizes (large ρ*), in contrast to the gyro-Bohm scaling obtained via local (flux tube) simulations. The results discussed here are obtained via linear, local simulations, carried out for fixed radial plasma parameter profiles. As such they represent an upper limit of the instability growth rate. When profile relaxation is not included, growth rates calculated via linear gyrokinetic simulations have been observed to be much less sensitive to radial system size^39^ and ρ*. Once nonlinear simulations become available, possible scaling differences between local and global gyrokinetic simulations in FRC geometry, characterized by relatively large ρ*, will need to be investigated, and results will be compared to fully kinetic calculations.

GTC linear stability calculations for the FRC core have been carried out for toroidal wavenumbers *k*_*θ*_*ρ*_*e*_≤0.3. The most important result of these calculations is that no unstable low frequency drift modes (*ω*<*ω*_ci_) have been detected for realistic normalized density and ion/electron temperature gradients (here *ω*_ci_ is the ion cyclotron frequency). In addition, GTC calculations have also confirmed that interchange modes (*k*_||_=0) are stable. Therefore, in agreement with experimental data, no unstable ion modes are revealed in the FRC core. Core stability is attributed to FLR effects, the radial gradient in the magnetic field (∇*B* directed oppositely to ∇*p*), and the short field-line connection length (short-circuit effect). The latter effect restricts the k_||_ spectrum such that even the lowest parallel wavenumbers in the drift wave spectrum are subject to electron Landau damping. To confirm the role of this effect, calculations have been carried out for an artificially elongated FRC configuration, and instability has been recovered for elongation factors above five. In addition, further efforts are presently under way to resolve the electron mode physics at high normalized wavenumber, and to assess the stability of high frequency, short scale modes up to the Lower Hybrid frequency range.

Extensive GTC linear stability calculations have also been carried out for the FRC SOL. The parameters used in the SOL simulation are *R*=0.27 m, *R*_s_=0.38 m, *n*_e_=2 × 10^19^ m^−3^, *T*_i_=200 eV, *T*_i_/*T*_e_=5. *Z*_eff_=1.5 is used for the effective charge state, as indicated experimentally via visible Bremsstrahlung measurements. It is known that in tokamak/toroidal simulations the inclusion of proper sheath boundary conditions is paramount to the accuracy of a numerical SOL description and that this is a significant source of uncertainty. A fully self-consistent treatment of the dynamics parallel to the magnetic field would require sheath boundary conditions in the axial direction in the FRC SOL also. However simpler periodic boundary conditions are appropriate in the axisymmetric FRC case within the local, electrostatic gyrokinetic approximation used here, as the relevant mode frequencies are substantially higher than the electron transit frequency. Periodic boundary conditions are used for the perturbed plasma density and electrostatic potential, and the particle motion in the toroidal direction and in the parallel direction at *z*=±*L*_c_. The axial boundary *L*_c_ is located outside the confined FRC region (*L*_c_=2–4 m versus *L*_FRC_≤1 m, where 2*L*_FRC_ is the axial length of the (closed flux surface) FRC plasma). Even with electrostatic end plate biasing the sheath impedance is still moderately large as the parallel electron current is below the ion saturation current and very small compared to the thermal electron current (electron saturation current). The magnetic field lines are fixed (tied) in the electrostatic approximation. Radial electron temperature 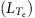, ion temperature 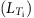 and density gradient scale lengths (*L*_*n*_) are assumed equal 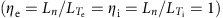 here and in most of the GTC runs executed so far. Experimentally, 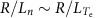 within the available resolution of the electron temperature measurements via multi-channel Thomson scattering^40^, once SOL parallel transport has established a connection to the axial boundary, as discussed below in more detail. In the simulations, the FRC SOL is close to collisionless with respect to ion-ion collisions, and collisional with respect to electron-electron and electron-ion collisions 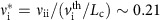, 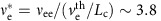 and 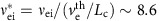, where *ν*_ee_, *ν*_ii_ and *ν*_ei_ are the electron-electron, ion-ion and electron-ion collision frequencies, 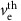 and 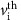 are the electron and ion thermal velocities, and 2*L*_c_ is the field line length in the SOL.

**Figure 6:**
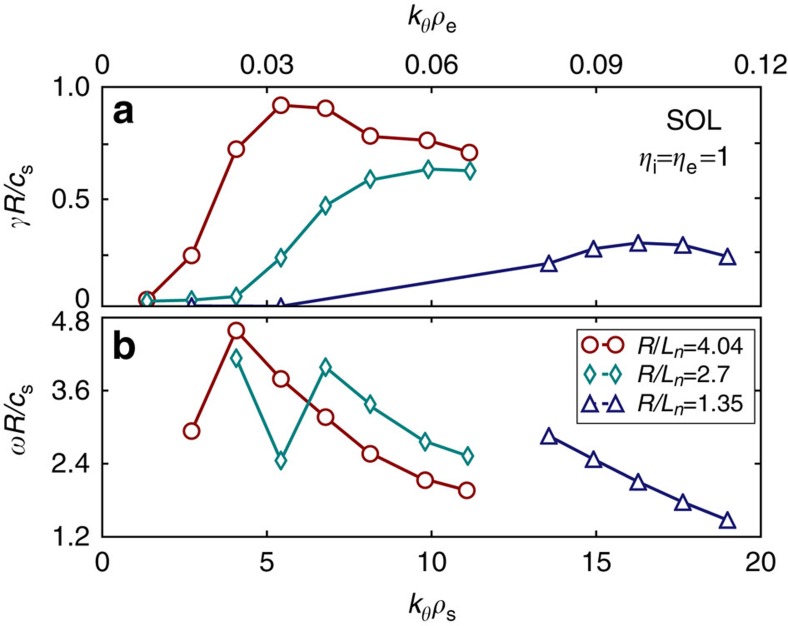
Linear instability growth rate and frequency in the scrape-off layer. (**a**) Normalized linear instability growth rate from an electrostatic flux tube calculation using the Gyrokinetic Toroidal Code (GTC)^20^,^21^,^35^ of scrape-off layer (SOL) modes versus normalized toroidal wavenumber *k*_*θ*_*ρ*_s_. The simulation flux tube radius (at the axial midplane) is *r*/*R*_s_∼1.3. The ratio of density gradient scale length to electron and ion temperature scale lengths is unity 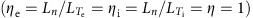. Results for different driving gradients are shown. With larger driving gradient, instability extends to lower normalized wavenumber. (**b**) Normalized frequency of scape-off layer (SOL) modes versus normalized toroidal wavenumber for the same simulation parameters.

**Figure 7:**
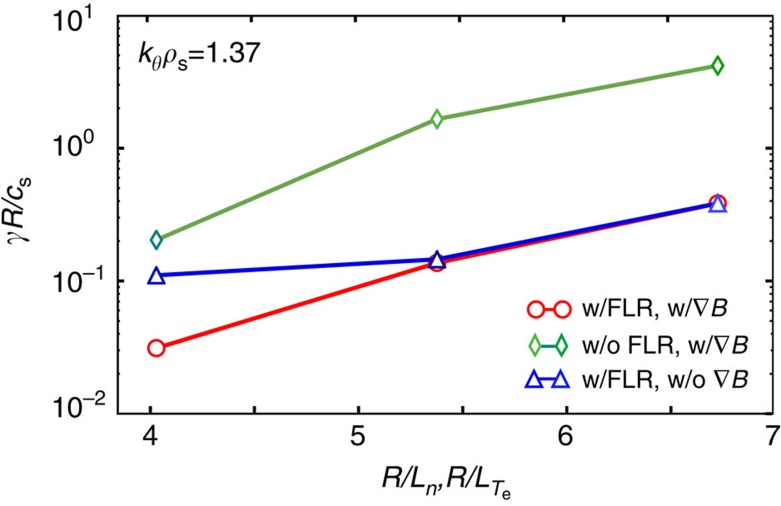
Effect of finite Larmor radius and magnetic field gradient on stability. The figure shows the linear instability growth rate for the scrape-off layer for three cases, where Finite Larmor radius effects and the radial magnetic field gradient have been selectively removed in the gyrokinetic stability calculations. Other simulation parameters are as stated previously. Finite Larmor radius effects are found to reduce the scrape-off layer linear instability growth rate very substantially, by about an order of magnitude. The comparison shown in the figure has been obtained by artificially removing Finite Larmor radius terms in the gyrokinetic equations. In a separate set of calculations the magnetic field gradient ∇*B* has been neglected. The figure shows that the magnetic field gradient also has a stabilizing influence at low driving strength.

In the present simulation, with gyrokinetic ions and drift-kinetic electrons, linear normalized growth rates *γR*/*c*_s_ are calculated for a range of normalized density and temperature gradient scale lengths. For the SOL simulations, the normalization factor *R*/*c*_s_=2.51 × 10^−6^ s. The flux tube radius used in the simulation, at the axial midplane, is *r*/*R*_s_∼1.3. It is remarkable that the growth rate spectrum (Fig. 6a) shows only unstable modes for 1.5≤*k*_*θ*_*ρ*_s_≤20 with toroidal wavelengths of the order of and smaller than the ion Larmor radius. The prevalent poloidal mode numbers (along the magnetic field lines) are found to be *m*=0–3. Figure 6b shows the frequencies of the unstable linear modes versus normalized wavenumber, indicating propagation in the positive (electron diamagnetic) direction. For realistic driving gradients, the calculated frequencies are in between the ion transit frequency and the ion diamagnetic drift frequency. Benchmarking calculations performed by selectively removing gyro-averaging and the magnetic field gradient in the gyrokinetic solver indicate clearly that both FLR effects and ∇*B* are strongly stabilizing^41^. This is demonstrated in Fig. 7. Removing gyro-averaging (FLR effects) results in an increase of the normalized growth rate by almost an order of magnitude for realistic temperature and density gradients. Selectively removing ∇*B*, in contrast, affects the growth rate significantly only at low driving strength *R*/*L*_*n*_.

**Figure 8:**
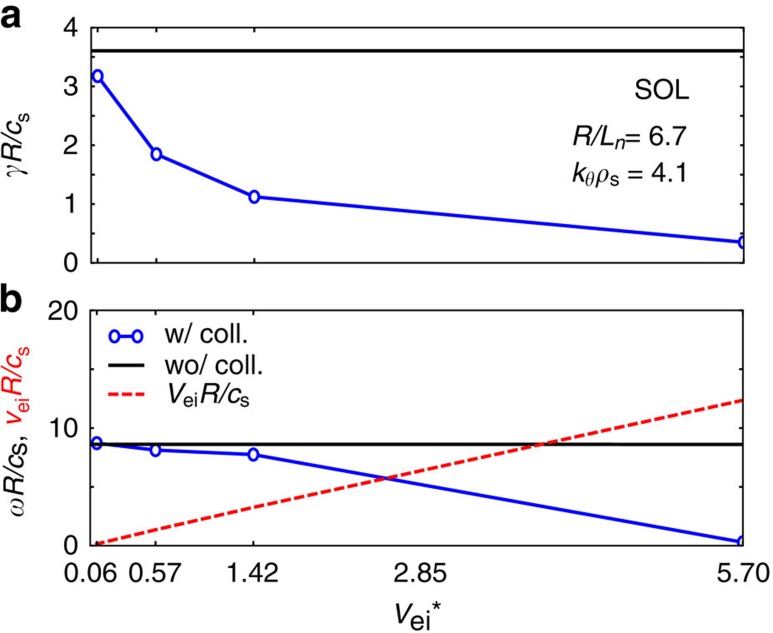
Effect of scrape-off layer collisionality on stability. The effect of including finite collisionality in the SOL linear growth rate calculations is demonstrated in this figure, for a sample toroidal wavenumber *k*_*θ*_*ρ*_s_=4.1. The normalized electron collisionality is defined as 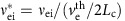, where *ν*_ei_ is the electron-ion collision rate, and *L*_c_ is the distance from the midplane to the axial boundary in the scrape-off layer (fieldline length=2*L*_c_). Other simulation parameters are as stated above. (**a**) normalized linear growth rate versus collisionality in comparison to the collisionless case; (**b**) normalized frequency as a function of collisionality. Collisions are seen to have a stabilizing effect and reduce the normalized growth rate substantially. The frequency of the mode investigated here is also greatly reduced with increasing electron-ion collision rate.

The effect of including finite collisionality in the linear growth rate calculations is demonstrated in Fig. 8, for a sample toroidal wavenumber *k*_*θ*_*ρ*_s_=4.1. The normalized electron collisionality is defined as 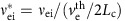, where *ν*_ei_ is the electron-ion collision rate, and 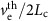 is the bounce frequency. *L*_c_ is the distance from the midplane to the simulation axial boundary (fieldline length=2*L*_c_) in the SOL. Collisions are seen to have a stabilizing effect and reduce the normalized growth rate substantially. The frequency of the mode investigated here is also greatly reduced with increasing collision rate.

Quantitative comparisons with the measured turbulence wavenumber spectrum will require nonlinear gyrokinetic simulations. Nonlinear effects, including three-wave interaction, are expected to lead to forward cascading of the wavenumber spectrum towards viscous scales, and inverse cascading towards low toroidal wavenumbers where large-scale zonal flows may be excited via three-wave interaction. Hence the expected saturated spectrum can be broadened or shifted substantially compared to the linear growth rate spectrum. The accessible wavenumber range in the measured saturated SOL density fluctuation spectrum (Fig. 4b) however clearly points towards an instability source at low toroidal wavenumber, in the range where the calculated linear growth rate spectrum is peaked.

### Critical SOL density gradient

**Figure 9:**
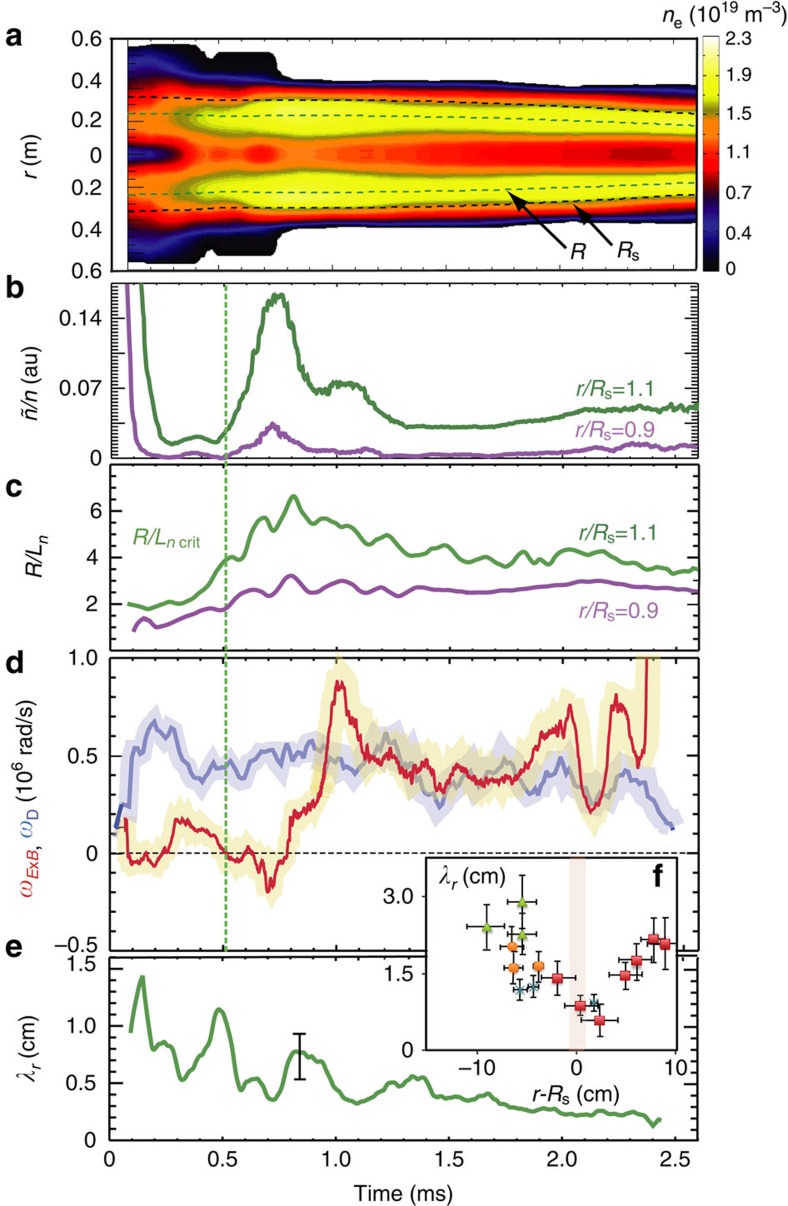
Evolution of plasma density fluctuation level and E × B shearing rate. (**a**) Time evolution of the plasma density profile shown as a contour plot. The null-field radius *R* (corresponding to the radius of highest plasma density) and the field reversed configuration (FRC) excluded flux radius *R*_s_ are indicated by dashed lines. (**b**) density fluctuation level *ñ*/*n* in the scrape-off layer (SOL) and in the closed flux surface region inside the field reversed configuration (FRC) separatrix. (**c**) normalized radial density gradient in the SOL and inside the separatrix. (**d**) **E** × **B** shearing rate *ω*_E × B_ and turbulence decorrelation rate *ω*_D_ in the SOL. The **E** × **B** shearing rate is shown to exceed the turbulence decorrelation rate after *t*∼1 ms; this condition is a prerequisite for the sheared flow to substantially reduce turbulence. (**e**) the time evolution of the radial turbulence correlation length in the shear flow region several centimeters outside the separatrix shows a gradual reduction indicating reduced radial extent of turbulent structures/eddies. (**f**) insert: radial profile of the radial turbulence (density fluctuation) correlation length, averaged from 1.2 to 2 ms. A dip in the correlation length just outside the FRC separatrix is indicative of the formation of a radial transport barrier. The error bars in Fig. 9 (**e**) and (**f**) represent the standard deviation (s.d.) of the measurements.

We now examine the interaction of the plasma density gradient and the turbulence dynamics in more detail. Figure 9a shows a contour/cross-sectional plot of the plasma density evolution during a C-2 discharge (#36691). The null-field radius *R* (corresponding to the radius of highest plasma density) and the FRC excluded flux radius *R*_s_ are indicated by dashed lines. The evolution of the measured rms density fluctuation level *ñ*/*n* is given in Fig. 9b both for the SOL and for the FRC core plasma. The value of *ñ*/*n* is relatively low for 0.2 ms<*t*<0.4 ms after the initial high turbulence period likely related to FRC translation and merging. The normalized radial density gradient (Fig. 9c) increases substantially during the first 1 ms, in particular in the SOL. We attribute this effect to parallel loss that dissipates/evacuates the SOL plasma produced during FRC formation and merging. The characteristic parallel loss time *τ*_∥_, for the case of collisionless ions can be estimated via the pitch angle scattering time *τ*_∥_ into the mirror loss cone *τ*_∥_=*τ*_∥_log_10_(Λ_m_) (ref. 42), where Λ_m_ is the primary mirror ratio. This estimate is expected to hold when the pitch angle scattering time of mirror-trapped ions exceeds the collisional parallel mirror confinement time^43^ *τ*_∥c_=*L*_SOL_Λ_m_/2(0.3*c*_s_)∼0.5 ms. Here, *L*_SOL_ is the distance from the FRC midplane to the axial endplates. For typical SOL parameters in C-2, *τ*_∥_∼*τ*_∥c_. The observed depletion timescale of the SOL plasma (∼0.7 ms) is comparable to *τ*_∥c_ and *τ*_∥_. At higher ion temperature the pitch angle scattering time is expected to set the parallel loss time. Within the available measurement resolution, the radial ion and electron temperature gradients are not observed to change substantially as the density gradient evolves and steepens. The electron temperature gradient scale length 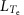 (from Thomson scattering) is similar to *L*_*n*_ after 1.2 ms within diagnostic uncertainty.

As shown in more detail in Fig. 9c, the initial increase in SOL fluctuation level for *t* >0.4 ms occurs as the density gradient exceeds a critical level or instability threshold^44^,^45^. The density gradient then relaxes somewhat during the remainder of the discharge but remains higher than the initial critical value. The SOL fluctuation level also decreases and returns to levels slightly above the early minimum.

We explain this behaviour by an upshift of the critical gradient in the presence of sheared **E** × **B** rotation analogous to the upshift in ion temperature gradient observed in gyrokinetic simulations of tokamak plasmas^46^. It is well known from tokamak experiments as well as linear plasma devices that sheared **E** × **B** flow strongly affects the growth and radial correlation of turbulent eddies, if the **E** × **B** shearing rate exceeds the ambient turbulence decorrelation rate, as discussed in detail elsewhere^47^. Figure 9d shows the evolution of the **E** × **B** shearing rate, determined via DBS from two radially adjacent probing radii, according to 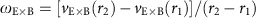 together with the turbulence decorrelation rate *ω*_D_. The decorrelation rate clearly exceeds the flow shearing rate initially, so that the **E** × **B** shear cannot quench or reduce the turbulence via eddy shearing/decorrelation^47^. Later in the discharge, at *t*∼1 ms, the **E** × **B** shear due to plasma gun biasing^26^,^27^ increases significantly. The shearing rate transiently exceeds the turbulence decorrelation rate *ω*_D_ and then remains close to *ω*_D_. Further evidence for the effect of **E** × **B** shear is provided by a decrease in the radial turbulence correlation length *λ*_*r*_ (Fig. 9e), which decreases steadily throughout the discharge. A radial profile of *λ*_*r*_ shows a distinct dip just outside the separatrix at the location of maximum shear (see insert, Fig. 9f). This is the first observation of a radial transport barrier in an FRC (with entirely different magnetic geometry compared to a tokamak) formed at/outside the separatrix on open field lines. In contrast, tokamak transport barriers form inside the separatrix in the closed flux surface region^48^.

**Figure 10:**
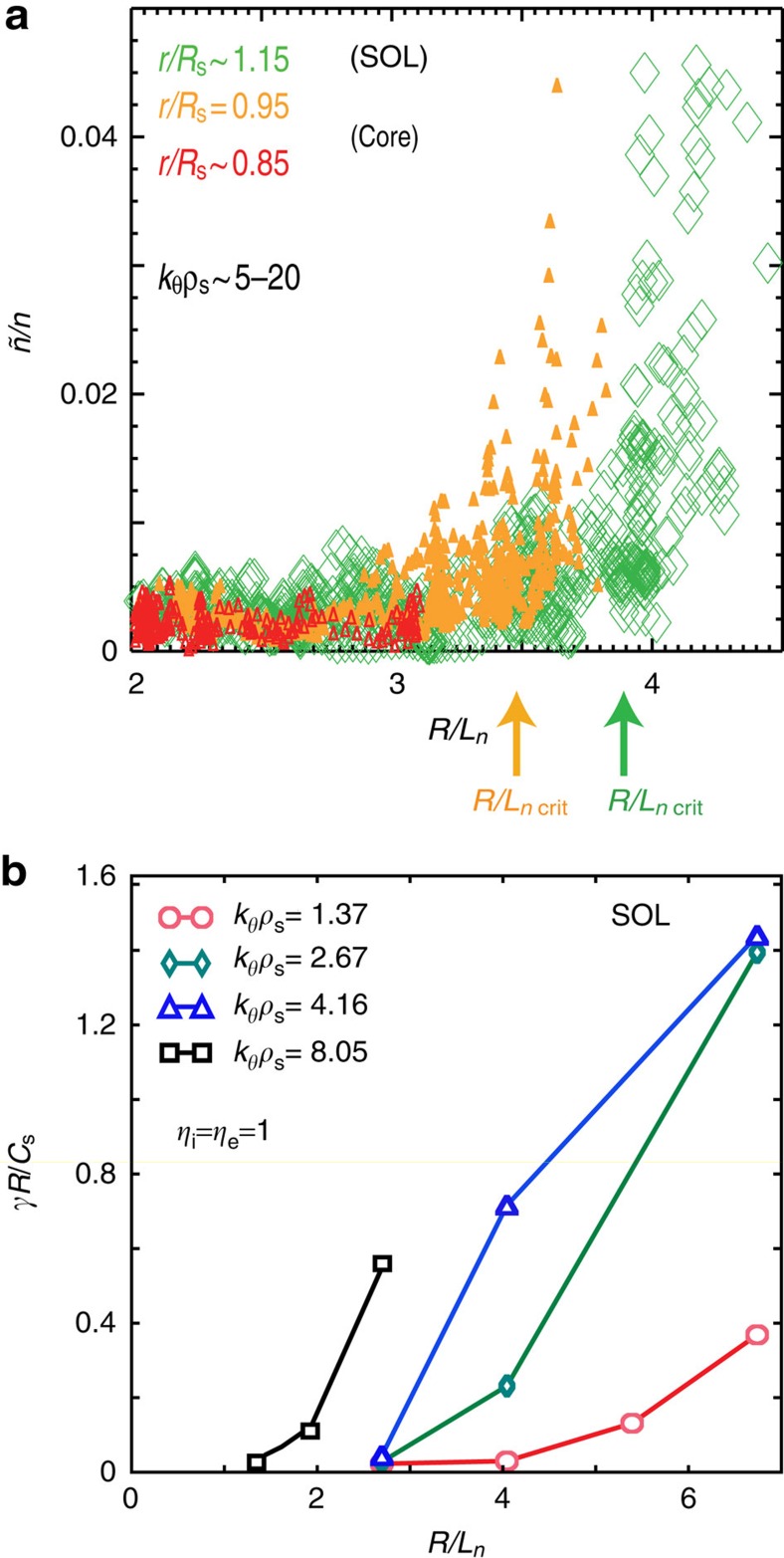
Density fluctuation level and calculated instability growth rate versus normalized density gradient. (**a**) Measured fluctuation level *ñ*/*n* versus the normalized density gradient in the field-reversed configuration (FRC) core (in red), near the separatrix (*r*/*R*_s_∼0.95, orange), and in the scrape-off layer (SOL), (*r*/*R*_s_∼1.15, green); the data sets taken near the separatrix and in the SOL show a clear increase at a critical density gradient. (**b**) calculated linear growth rate from the Gyrokinetic Toroidal Code (GTC) versus normalized density gradient *R*/*L*_n_ in the SOL (*r*/*R*_s_=1.2, *η*=1), for different normalized toroidal wavenumbers as indicated in the figure. Characteristic perpendicular fluctuation scale lengths observed in the C-2 FRC are in between the ion and electron Larmor radii, corresponding to the electron mode regime. The linear instability threshold for *k*_*θ*_*ρ*_s_=1.37 and *k*_*θ*_*ρ*_s_=2.67 is similar to the (nonlinear) critical gradient observed in the fluctuation level measurements.

To elucidate the role of the critical density gradient, Fig. 10a shows a plot of the measured fluctuation level versus the local density gradient. Well into the FRC core (*r*/*R*_s_=0.85), the density gradient remains moderate (*R*/*L*_*n*_≤3.1), fluctuation levels are very low and no nonlinear increase of *ñ*/*n* with gradient has been observed. Just inside the separatrix (*r*/*R*_s_=0.95), a critical gradient around *R*/*L*_*n*_∼3.5 is found experimentally. In the SOL, a pronounced critical gradient *R*/*L*_*n*_^crit^∼3.8–4 is measured during the early discharge phase (*t*<0.6 ms). For the SOL the linear GTC simulation yields a linear threshold *R*/*L*_*n*_∼2.7 (Fig. 10b) for toroidal wavenumbers 2.7≤*k*_*θ*_*ρ*_s_<4.2 within the most unstable part of the growth rate spectrum. The measured SOL critical gradient is somewhat higher than the calculated linear threshold, as expected if local flow shear resulting from the equilibrium radial electric field or from zonal flow components effectively upshifts the nonlinear critical gradient. This effect is typically observed in nonlinear gyrokinetic simulations^45^,^46^. A lower linear threshold is found here for high toroidal wavenumber; however, the energy density in the higher-*k* part of the spectrum is substantially lower, and the associated radial transport rates are expected to be low. Importantly, a further upshift of the measured SOL density gradient is clearly detected experimentally for *t* >1.2 ms when the **E** × **B** shear is fully developed (Fig. 9c). A moderately large SOL critical gradient, as measured here, is favourable for achieving a sufficiently narrow SOL for reactor-like FRC parameters. This is significant, as a narrow SOL minimizes the required device radius and is advantageous for achieving a compact fusion core. The increased parallel heat flux in a narrow SOL can be exhausted in an axisymmetric FRC via radial magnetic flux expansion, and the heat load to plasma facing components can be kept within safe limits. In contrast, the flux expansion in tokamak poloidal divertors is geometrically limited, and a narrow SOL is problematic as it increases the divertor peak heat load.

## Discussion

In conclusion, we have presented experimental evidence that ion-scale fluctuations with toroidal scale length on the order of or larger than the ion gyroradius are stable in the core of a large FRC configuration. The observed ion-scale drift-wave/interchange stability is a new discovery that is expected to hold generically in FRCs, independently of the specific C-2 machine parameters explored here. This result is highly encouraging because both ion and electron thermal transport are most strongly driven by low-*k* (ion-scale) fluctuations.

Only weak unstable electron-mode fluctuations with toroidal scale lengths smaller than the ion gyroradius are observed in the C-2 FRC core plasma, leading to an inverted toroidal wavenumber spectrum for *k*_*θ*_*ρ*_s_≤7. The observed absence of low-k fluctuations is ascribed to Finite Larmor radius effects due to the large bulk ion orbits in the FRC core. In addition, the radially increasing magnetic field (with ∇*B* opposing ∇*p*), and the short field line connection length contribute to stability. Due to the short connection length, drift wave parallel wavenumbers are constrained to *k*_∥_>0.02 cm^−1^, and the corresponding mode spectrum is subject to electron Landau damping. In addition, low *k*_*θ*_ interchange modes (*k*_∥_=0) are also found to be stable in the FRC core. These findings may have general implications for ion and electron thermal confinement in hot ion, high beta plasmas, including collisionless astrophysical plasmas.

Low levels of ion-scale turbulence have also been observed in high confinement mode (H-mode) plasmas in spherical tokamaks^49^ and in certain H-mode plasmas in larger aspect ratio devices. However, in tokamaks the observed reduction in ion-scale core turbulence results from strong shear in the toroidal rotation, leading to long-wavelength turbulence quench via **E** × **B** shear, in combination with magnetic shear^48^. In contrast, there is only weak **E** × **B** flow shear in the FRC core in our experiment, and the observed absence of ion-scale turbulence is due to effects directly suppressing linear instability growth as discussed above.

There is another important difference between FRCs and tokamak plasmas with respect to the minimum achievable ion thermal transport. In tokamaks, due to toroidicity, neoclassical ion transport is the irreducible minimum. At the same magnetic field and collisionality, neoclassical transport still substantially exceeds classical transport. FRCs, on the other hand, potentially can achieve classical ion transport due to axisymmetry.

FRC instability and transport physics is substantially different from tokamaks, and global FRC energy confinement in the presence of classical ion transport is determined by the electron channel, opening the prospect of radically different FRC electron thermal confinement scaling compared to conventional tokamaks. The electron energy confinement scaling observed in the MAST and NSTX spherical tokamaks, however, has already shown a favourable scaling with decreasing collisionality^49^,^50^,^51^. The confinement time varies approximately inversely with the normalized electron collisionality which scales as 1/*T*_e_^2^.

Ion and electron-scale turbulence with higher density fluctuation levels is observed experimentally in the SOL region just outside the FRC separatrix, where an exponential wavenumber spectrum is measured. Linear gyrokinetic simulations show unstable SOL modes in the wavenumber range 1.5≤*k*_*θ*_*ρ*_s_≤20 propagating in the electron diamagnetic direction, with frequencies well above the ion and electron transit frequencies, and below the ion diamagnetic frequency. Benchmarking calculations demonstrate directly the stabilizing effects of both finite Larmor radius (FLR) and the magnetic field gradient for the SOL modes, with FLR effects reducing the growth rate by more than one order of magnitude. Collisions are also found to reduce the linear instability growth rate substantially in the SOL. Experimentally, the SOL turbulence amplitude increases nonlinearly at a critical density gradient *R*/*L*_*n*_∼4, somewhat above the linear instability threshold as calculated from linear gyrokinetic simulations. The critical gradient is sufficiently large so that the SOL in a reactor-relevant FRC plasma would remain reasonably narrow and not unduly increase the required device radius.

For the first time to the best of our knowledge, evidence for sustained turbulence reduction/gradient control via **E** × **B** flow shear has been found in FRC geometry. In contrast to tokamak edge and core transport barriers, the FRC transport barrier is localized just outside the separatrix on open magnetic field lines, which terminate in the divertor and are actively biased in a controlled fashion. The prospect of active control of turbulence and radial transport across the FRC separatrix, via manipulating the **E** × **B** shear by electrostatic biasing in the divertor far away from the hot core plasma, opens up the possibility of reducing radial thermal and particle loss from the confined FRC region, and hence advancing towards long-pulse FRC operation with reduced fuelling and auxiliary heating/current drive sustainment requirements.

## Methods

### The C-2 field-reversed configuration

The C-2 FRC plasma is created via injecting and merging of two preformed, compact high-*β* plasmoids into a central confinement chamber with radius *R*=0.7 m and length *L*=4.5 m (Fig. 1a), with an external solenoidal magnetic field *B*_e_=0.05–0.14 T, as described in detail elsewhere^17^,^18^,^23^. The injected plasmoids are produced in the formation sections (Fig. 1a) with an ion temperature of 25–50 eV before merging, and are accelerated to high axial (parallel) velocity. The axial plasmoid velocity at the transition from the formation sections to the central confinement chamber is∼2.5 × 10^5^ m s^−1^, corresponding to a (directional) parallel energy of ∼1 keV that is transformed into thermal energy upon merging, likely via shock heating. Typical line-averaged deuterium plasma densities of the merged FRC plasma in the experiments described here are 2–4 × 10^19^ m^−3^, and the ion and electron temperatures in the FRC core are *T*_i_∼400–600 eV and *T*_e_∼80–130 eV. Toroidally injected neutral beams (hydrogen, beam voltage *V*_B_=20 kV and total beam power, *P*_B_∼4 MW) are applied to heat and sustain the FRC plasma and to extend the FRC pulse length to∼5 ms. The absorbed neutral beam power is 2.3–2.7 MW throughout the first 2 ms of the discharge and slowly declines afterwards as the FRC gradually contracts and NBI shine-through increases. As *T*_i_>*T*_e_, NBI beams predominantly heat electrons (via collisional exchange) and electron thermal transport dominates the beam power absorption. Fast ion lifetimes are consistent with classical slowing-down via Coulomb collisions^18^. With high neutral beam power deposition (*P*_abs_=2.5 MW) the global FRC energy confinement time is doubled compared to no-beam reference cases^18^. The (closed flux surface) FRC plasma in C-2 is surrounded by an (open fieldline) mirror-confined SOL plasma with primary mirror ratio *Λ*_m_∼3. In addition to the primary mirror, secondary mirror plugs are installed (shown in Fig. 1) with a mirror ratio Λ_p_=7–10. The SOL plasma is terminated axially on (floating or biased) metallic electrodes located in divertor chambers at a distance of 8.8 m from the machine axial midplane. In the SOL the thermal ion Larmor radius, averaged along the field line length, is *ρ*_i_∼2–3 cm. Closer to the null field radius in the FRC core the thermal ion Larmor radius is substantially larger and can reach *ρ*_i_≥10 cm. The electron Larmor radius is almost always much smaller than the radial temperature/density scale lengths (*ρ*_e_∼2 × 10^−2^–10^−1^ cm).

### Plasma guns

Annular washer plasma guns^25^ (inner diameter 0.11 m, outer diameter 0.13 m, located on axis in the divertor chambers as indicated in Fig. 1a) are used to produce a warm plasma with *T*_e_∼30 eV, *T*_i_∼100 eV in the divertor. The local magnetic field at the gun location is ∼0.5 T, produced by an internal solenoid integrated with the plasma gun assembly. The washer guns have anode-cathode voltages of∼0.5–1.0 kV, with typical discharge current ∼10 kA. The plasma guns are electrically floating with respect to the vacuum chamber, producing a negative (inwards pointing) radial electric field transmitted to the SOL region just outside the FRC separatrix^26^,^27^. In a different biasing approach applied more recently, the co-axial endplate inside the gun annular anode is biased negatively at 0.5–1.0 kV with respect to the innermost annular divertor biasing electrode, with an arc discharge initiated simultaneously between the plasma gun cathode and anode.

### Additional details on diagnostics

A six-channel Doppler Backscattering (DBS)^28^,^29^ system is used for measurements of the density fluctuation level, and of the toroidal **E** × **B** velocity (Fig. 2a,b). Six collinear microwave beams, at tunable frequencies 26 GHz<*f*<65 GHz, are launched via two lensed horn antennas and quasi-optically transmitted to the plasma at an oblique angle ζ in the toroidal plane^30^. A polarizer/beam combiner and an adjustable parabolic mirror are used to combine the different frequency bands and focus the combined Gaussian beams into the plasma. Frequencies in the range 26–40 GHz are launched in X-mode polarization, and frequencies above 40 GHz are launched in O-mode polarization. The backscattered radiation is detected via the same horn antennas (monostatic beam optics), at an oblique angle in the toroidal plane. Figure 2a,b illustrate the beam path and scattering geometry for one frequency. The rms density fluctuation level (0.5≤*k*_*θ*_*ρ*_s_≤40), toroidal **E** × **B** velocity and turbulence decorrelation rate near the FRC midplane are evaluated from the intensity and Doppler shift of the backscattered signals.

Backscattering by plasma density fluctuations occurs preferentially near the plasma cutoff layer^28^, according to the selection rules *k*_S_=−*k*_I_=*k*_*θ*_/2 and *ω*_S_=*ω*_I_−*ν*_t_*k*_*θ*_, where the indices I and S denote the incident and backscattered wave, *ν*_t_=*ν*_E × B_+*ν*_ph_ is the toroidal turbulence advection velocity (*v*_ph_ is the turbulence phase velocity in the plasma frame and can be neglected here), and *k*_*θ*_ is the probed resonant density fluctuation toroidal wavenumber. Variation of the toroidal launch angle allows acquiring the density fluctuation level versus toroidal wavenumber. The toroidal wavenumber resolution is primarily determined by the toroidal plasma curvature and the width of the Gaussian probing beams^52^. The probed radial wavenumber is *k*_*r*_∼0 as the beams propagates toroidally near the plasma cutoff layer. The wavenumber *k*_*θ*_ and the probed radii *r* in the laboratory frame are calculated using the GENRAY^31^ ray tracing code. Ray tracing calculations are based on high time resolution (10 μs) radial electron density profiles (Fig. 1(c)) reconstructed from a six channel CO_2_ laser interferometer^53^ located in the FRC axial midplane. All calculated DBS probing positions are mapped to the axial FRC midplane. For typical C-2 plasma parameters the X-mode and O-mode cutoff layer positions differ only very slightly as the plasma frequency is much larger than the electron cyclotron frequency.

Fluctuation levels extracted from DBS are calibrated here in an approximate fashion by comparison with (line-integrated) Far Infrared Scattering data^54^ obtained from an FIR chord traversing the plasma tangentially to the separatrix. FIR measures a perpendicular wavenumber range *k*_⊥_*ρ*_s_∼0–4. We make the assumption here that the fluctuation spectrum is isotropic in the toroidal and radial direction. The largest contribution to the measured fluctuation amplitude for this FIR chord will come from the vicinity of the separatrix, where the density (and density gradient) is largest. Choosing a similar wavenumber range for the DBS measurements, a rough calibration for the DBS fluctuation levels can be obtained with a systematic maximum error of +40%, −50%.

### Data availability

The data that support the findings of this study are available from the corresponding author on request.

## Additional information

**How to cite this article:** Schmitz, L. *et al*. Suppressed ion-scale turbulence in a hot high-β plasma. *Nat. Commun.* **7,** 13860 doi: 10.1038/ncomms13860 (2016).

**Publisher's note:** Springer Nature remains neutral with regard to jurisdictional claims in published maps and institutional affiliations.

## References

[b1] Wesson J. Tokamaks 4th edn. Oxford University Press (2011).

[b2] Tuszewski M. Field-reversed configurations. Nucl. Fusion 28, 2033–3093 (1988).

[b3] Steinhauer L. C. Review of field-reversed configurations. Phys. Plasmas 18, 070501 (2011).

[b4] Rostoker N. . Colliding beam fusion reactor. Science 278, 1419–1423 (1997). 9367946 10.1126/science.278.5342.1419

[b5] Balbus S. A. & Hawley J. F. Instability, turbulence, and enhanced transport in accretion disks. Rev. Mod. Phys. 70, 1–53 (1998).

[b6] Schekochikin A. A. . Astrophysical gyrokinetics: kinetic and fluid turbulent cascades in magnetized weakly collisional plasmas. Astrophys. J. Suppl. 182, 310–377 (2009).

[b7] Maehara H. . Superflares on solar-type stars. Nature 485, 478–481 (2012). 22622572 10.1038/nature11063

[b8] Hoffman A. L. & Slough J. T. Field reversed configuration lifetime scaling based on measurements from the large s experiment. Nucl. Fusion 33, 27–38 (1993).

[b9] Naitou H., Kamimura T. & Dawson J. M. Kinetic effects on the convective plasma diffusion and the heat transport. JPSJ 46, 258–265 (1979).

[b10] Rosenbluth M. N., Krall N. & Rostoker N. Finite Larmor radius stabilization of ‘weakly' unstable confined plasmas. Nucl. Fusion Supplement Pt 1, 143–150 (1962).

[b11] Rostoker N. in *Physics of High Energy Particles in Toroidal Systems* (eds Tajima, T. & Okamoto, M.) 323 (American Institute of Physics, 1994).

[b12] Binderbauer M. & Rostoker N. Turbulent transport in magnetic confinement: how to avoid it. J. Plasma Phys. 56, 451–465 (1996).

[b13] McKenna K. F. . Particle confinement scaling in field-reversed configurations. Phys. Rev. Lett. 50, 1787–1790 (1983).

[b14] Carlson A. W. A search for lower-hybrid-drift fluctuations in a field-reversed configuration using CO_2_ heterodyne scattering. Phys. Fluids 30, 1497–1509 (1987).

[b15] Farengo R., Guzdar P. N. & Lee Y. C. Collisionless electron temperature gradient-driven instability in field-reversed configurations. Phys. Fluids B1, 2181 (1989).

[b16] Yu C. A. & Khvesyuk V. I. Electromagnetic drift instabilities in high-*β* plasma under conditions of a field-reversed configuration. Phys. Plasmas 17, 012105 (2010).

[b17] Binderbauer M. . Dynamic formation of a hot field reversed configuration with improved confinement by supersonic merging of two colliding high-β compact toroids. Phys. Rev. Lett. 105, 045003 (2010). 20867853 10.1103/PhysRevLett.105.045003

[b18] Binderbauer M. . A high performance field-reversed configuration. Phys. Plasmas 22, 056110 (2015).

[b19] Gupta S. . Transport studies in high performance field-reversed configuration plasmas. Phys. Plasmas 23, 052307 (2016).

[b20] Fulton D. P., Lau C., Holod I., Lin Z. & Dettrick S. Gyrokinetic particle simulation of a field reversed configuration. Phys. Plasmas 23, 012509 (2016).

[b21] Fulton D. P. . Gyrokinetic simulation of driftwave instability in field-reversed configuration. Phys. Plasmas 23, 056111 (2016).

[b22] Mase A. . Ambipolar potential effect on drift-wave mode in a tandem-mirror plasma. Phys. Rev. Lett. 64, 2281–2284 (1990). 10041634 10.1103/PhysRevLett.64.2281

[b23] Guo H. Y. . Formation of a long-lived hot field reversed configuration by dynamically merging two colliding high-β compact toroids. Phys. Plasmas 18, 056110 (2011).

[b24] Galeotti L., Barnes D. C., Ceccherini F. & Pegoraro F. Plasma equilibria with multiple ion species: equations and algorithm. Phys. Plasmas 18, 082509 (2011).

[b25] Akhmetov T. D. . Experiments with dense plasma in the central solenoid of AMBAL-M. Trans. Fusion Sci. Technol. 43, 58–62 (2003).

[b26] Tuszewski M. . Field reversed configuration confinement enhancement through edge biasing and neutral beam injection. Phys. Rev. Lett. 108, 255008 (2012). 23004613 10.1103/PhysRevLett.108.255008

[b27] Tuszewski M. . A new high performance field reversed configuration operating regime in the C-2 device. Phys. Plasmas 19, 056108 (2012).

[b28] Peebles W. A. . A novel, multichannel, comb-frequency Doppler backscatter system. Rev. Sci. Instrum. 81, 10D902 (2010). 10.1063/1.3464266 21033934

[b29] Hillesheim J. C. . New plasma measurements with a multichannel millimeter-wave fluctuation diagnostic system in the DIII-D tokamak. Rev. Sci. Instrum. 81, 10D907 (2010). 10.1063/1.3466900 21033939

[b30] Schmitz L. . Multi-channel Doppler backscattering measurements in the C-2 field reversed configuration. Rev. Sci. Instrum. 85, 11D840 (2014). 10.1063/1.4891415 25430253

[b31] Smirnov A. P. & Harvey R. W. *The GENRAY ray tracing code.* Report COMPX-2000-01 (COMPX-X, 2001).

[b32] Hennequin P. . Fluctuation spectra and velocity profile from Doppler backscattering on Tore Supra. Plasma Phys. Controlled Fusion 46, S771–S779 (2006).

[b33] Schmitz L. . Reduced electron thermal transport low collisionality H-mode plasmas and the importance of TEM/ETG-scale turbulence. Nucl. Fusion 52, 023003 (2012).

[b34] Lin Z. . Turbulent transport reduction by Zonal Flows: massively parallel simulations. Science 281, 1835–1837 (1998). 9743492 10.1126/science.281.5384.1835

[b35] Lin Z. . Gyrokinetic Toroidal Code ∼GTC http://phoenix.ps.uci.edu/GTC/codes.php (2010).

[b36] Boozer A. H. Plasma equilibrium with rational magnetic surfaces. Phys. Fluids 24, 1999–2003 (1981).

[b37] Lin Z., Ethier S., Hahm T. S. & Tang W. M. Size scaling of turbulent transport in magnetically confined plasmas. Phys Rev. Lett. 88, 195004-1–195004-4 (2002). 12005641 10.1103/PhysRevLett.88.195004

[b38] Candy J., Waltz R. E. & Dorland W. The local limit of global gyrokinetic simulations. Phys. Plasmas 11, L25 (2004).

[b39] McMillan B. F. . System size effects on gyrokinetic turbulence. Phys. Rev. Lett. 105, 155001 (2010). 21230913 10.1103/PhysRevLett.105.155001

[b40] Deng B. H., Kinley J. S. & Schroeder J. Electron density and temperature profile diagnostics for C-2 field-reversed configuration plasmas. Rev. Sci. Instrum. 83, 10E339 (2012). 10.1063/1.4740263 23126997

[b41] Lau C. K. . in 57th Meeting of the American Physical Society, Division of Plasma Physics. Available at http://meetings.aps.org/Meeting/DPP16/Session/CP10.74 (2016).

[b42] Pastukhov V. P. Collisional losses of electrons from an adiabatic trap in a plasma with a positive potential. Nucl. Fusion 14, 3–6 (1974).

[b43] Lam K. L. . Mirror ratio scaling of axial confinement of a mirror-trapped collisional plasma. Phys. Fluids 29, 3433–3438 (1986).

[b44] Steinhauer L. C. Anomalous transport effects in magnetically-confined plasma columns. Phys. Fluids 21, 230–236 (1978).

[b45] Garbet X. Turbulence in fusion plasmas—key issues and impact on transport modeling. Plasma Phys. Control. Fusion 43, A251–A266 (2007).

[b46] Dimits A. M. . Scalings of ion-temperature-gradient-driven anomalous transport in Tokamaks. Phys. Rev. Lett. 77, 71–74 (1996). 10061774 10.1103/PhysRevLett.77.71

[b47] Biglari H., Diamond P. H. & Terry P. Influence of sheared poloidal rotation on edge turbulence. Phys. Fluids B 2, 1–4 (1990).

[b48] Burrell K. H. . Role of the radial electric field in the transition from L (low) mode to H (high) mode to VH (very high) mode in the DIII-D tokamak. Phys. Plasmas 1, 1536–1544 (1994).

[b49] Kaye S. M. . Confinement and local transport in the National Spherical Torus Experiment (NSTX). Nucl. Fusion 47, 499 (2007).

[b50] Valovic M. . Scaling of H-mode energy confinement with I_p_ and B_t_ in the MAST spherical tokamak. Nucl. Fusion 49, 075016 (2009).

[b51] Kaye S. M. . The dependence of H-mode energy confinement and transport on collisionality in NSTX. Nucl. Fusion 53, 063005 (2013).

[b52] Blanco E. . Doppler reflectometry studies using a two-dimensional full wave code. Plasma Phys. Control. Fusion 48, 699–714 (2006).

[b53] Gornostaeva O. . Two-color CO_2_/HeNe laser interferometer for C-2 experiment. Rev. Sci. Instrum. 81, 10D516 (2010). 10.1063/1.3478983 21033871

[b54] Deng B. H. . Far infrared laser polarimetry and far forward scattering diagnostics for the C-2 field reversed configuration plasma. Rev. Sci. Instrum. 85, 11D401 (2014). 10.1063/1.4884903 25430164

